# CuPc Passivation of a MAPbBr_3_ Single Crystal Surface

**DOI:** 10.1021/acs.jpcc.3c04209

**Published:** 2023-09-26

**Authors:** Ke Wang, Benjamin Ecker, Mingze Li, Jinsong Huang, Yongli Gao

**Affiliations:** † Department of Physics and Astronomy, University of Rochester, Rochester, New York 14627, United States; ‡ Department of Applied Physical Sciences, University of North Carolina at Chapel Hill, Chapel Hill, North Carolina 27599, United States

## Abstract

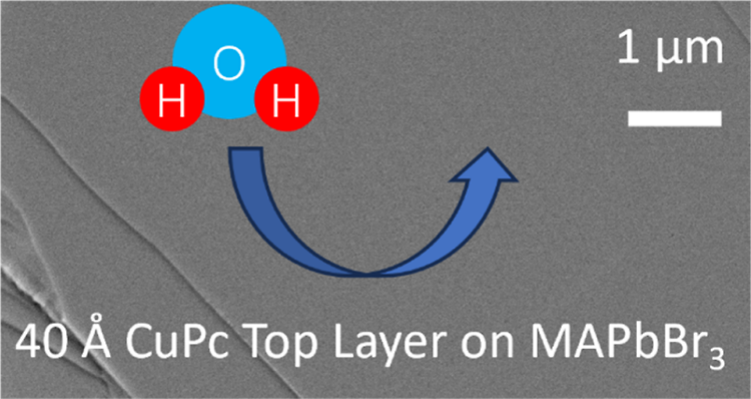

In this study, a facile passivation for methylammonium lead bromide (MAPbBr_3_) single crystals is reported. Stability against moisture and light remains the most critical demerit of perovskite materials, which is improved by depositing a 40 Å thick hydrophobic copper phthalocyanine (CuPc) layer on top of the cleaved perovskite surface. The water and light exposure processes were monitored with X-ray photoelectron spectroscopy with precise control of the exposure time and pressure. It is found that the CuPc top layer could protect the sample from moisture infiltration at a water exposure of 10^13^ L, while the nonpassivated sample started to degrade at 10^8^ L. During the light exposure, CuPc also slowed down the light-induced degradation, which is supported by the elemental ratio change of metallic lead and bromine. These results are further confirmed by the morphological comparison via scanning electron microscopy and focused ion beam.

## Introduction

Organic–inorganic hybrid perovskites are considered to be ideal candidates for next generation solar cells as the power conversion efficiency of perovskite solar cells (PSCs) experienced a rapid development from 3.3% in 2009 to 25.7% in 2022.^1–3^ Compared to its peers, methylammonium lead halide (MAPbX_3_) exhibits excellent optical and electrical properties, including high absorption coefficient, high charge carrier mobility, long carrier lifetimes, low trap density, and high photoluminescence quantum yield, which result in a strong light-harvesting ability and high efficiency in light emission and detection.^4–9^ These outstanding features of MAPbX_3_ have broadened its potential applications to solid state lasers, light-emitting diodes, photodetectors, and gas detectors.^10–13^ However, one of the biggest issues with this type of material is that it is vulnerable to almost all environmental factors such as heat, moisture, gas exposure, and light irradiation.^14–23^ A lot of techniques have been applied to enhance the stability of perovskites including solvent engineering, additive engineering, and passivation engineering.^24^ In particular, passivation techniques can decrease the efficiency loss by effectively reducing charge carrier recombination and ion migration. Abdi-Jalebi et al. reported that potassium iodide can fill the iodine vacancies and then suppress the ion migration process.^25^ Jiang et al. synthesized planar-structure PSCs with PbI_2_ passivation, achieving efficiency surpassing 21%, where moderate excess PbI_2_ could enhance charge separation and reduce the carrier recombination at the interface.^22^ Copper phthalocyanine (CuPc), a bright, crystalline, synthetic blue pigment, was first used as a p-type semiconductor in light-emitting diodes and organic solar cells for its outstanding carrier mobility and stability.^26,27^ It was introduced to PSCs as a hole transport layer (HTL), and Ke et al. fabricated PSCs with long-term thermal stability and an efficiency of 14.5% using CuPc as the HTL.^28^ Qu et al. further improved the efficiency of PSCs employing CuPc derivatives to 23% and maintained 96% of their initial efficiency after 3624 h of aging at 85 °C.^29^ Various works have been done focusing on the device level and showed that CuPc is an ideal HTL material with long-term thermal stability.^28–33^ However, the effects of CuPc passivation at the surface analytical level are rarely touched by the research community. Especially, as a hydrophobic material, its effects on the water and light stability of perovskite are yet to be investigated.

In this article, we report our systematic experimental investigation on the CuPc passivation effect of the in situ cleaved MAPbBr_3_ single crystal under water and light exposures at the surface level in an ultrahigh-vacuum (UHV) system. By depositing a 40 Å thick top layer of CuPc onto the MAPbBr_3_ single crystal, excellent protection against water and reduced light degradation were observed. X-ray photoemission spectroscopy (XPS) was used to monitor the chemical compositional changes of the perovskite sample. Water exposure was carefully controlled and measured in a Langmuir instrument (L, 1 L = 10^–6^ Torr·s). Scanning electron microscopy (SEM), along with focused ion beam (FIB), provided both surface and bulk morphological information on the single crystal. We found that for the CuPc-passivated sample, the surface had no oxygen signals after the total 10^13^ L exposure (equivalent to 200 h), while the pristine sample started to see a non-neglectable oxygen component at 10^5^ L, and it continued to grow with the water exposure. It shows that the water molecules were expelled from the sample surface by the CuPc layer, which protected the sample from water infiltration and water-induced degradation. In light exposure, the perovskite Pb decomposes into metallic Pb, and C, N, and Br decompose into volatile species and leave the surface. With CuPc passivation, 36.3% of total Pb was converted to metallic Pb after 44 h of light exposure, while the number increased to 64.0% for the pristine crystal. The Br ratio also saw a slower decay rate with the CuPc-passivated sample. Furthermore, the CuPc layer remained intact after water exposure, in contrast to the almost complete desorption observed after light exposure. SEM and FIB analyses showed that the passivated sample underwent minimal changes in both surface and bulk after water exposure, with only a mild surface roughening observed compared to the pristine sample after light exposure. The results indicate that CuPc is not only an ideal thermally stable HTL material but also can enhance the stability of perovskite against both water and light, which could potentially expand the range of applications for CuPc.

## Methods

The MAPbBr_3_ single crystal was prepared by a solution-processed antisolvent growth method, and the details can be found in ref (5). The single crystal sample used in this study has an average size of ∼5 × 5 × 4 mm. The sample was cleaved in situ to obtain a contamination-free pristine surface, and both the exposure and CuPc deposition processes were carried out in a vacuum system that is directly connected to the XPS analytical chamber. XPS was operated at 10 kV and 10 mA with a monochromatic Al Kα source (1486.6 eV). The energy resolution is about 0.6 eV with a pass energy of 148.64 eV. XPS was calibrated by determining the zero of the binding energy (BE) scale from a measurement of the Fermi edge of Au, and the Au core levels were within 0.1 eV from the published results.^34^ Once the as-cleaved pristine sample was measured with XPS, it was then transferred to an evaporation chamber for CuPc passivation. CuPc powder was purchased from Sigma-Aldrich and loaded in a tantalum boat for thermal evaporation. The evaporation rate was kept at 1 Å/min, and the thickness was monitored with a quartz crystal monitor.

For water exposure, the steps prior to 10^5^ L were conducted in the analytical chamber, and the remaining steps were conducted in the exposure chamber with a base pressure of 1 × 10^–6^ Torr. The water vapor was supplied with a distilled water tube that was connected to both the XPS chamber and the exposure chamber. For light exposure, it was stored in the analytical chamber with a base pressure of 1 × 10^–10^ Torr for the entire exposure period. The excitation source was a 408 nm continuous wave (CW) laser with an intensity of 7.65 mW/mm^2^, which is approximately seven times the sun intensity (1 mW/mm^2^). It was attached to a view port of the chamber, and the illuminated spot was positioned at the center of the surface. After each step, the sample was immediately transferred to the XPS chamber for the XPS measurements. A microscope was mounted onto the analytical chamber to monitor the XPS measuring spot, ensuring that each measurement was taken at the same location.

After exposure, the sample was transferred to a Zeiss Auriga SEM–FIB system in a vacuum-sealed desiccator for morphological and depth profiling. The accelerating voltage of the SEM was set to 5 kV to minimize the damage from the electron beam. FIB was used to mill a 5–10 μm deep trench to investigate the effects on the bulk part. The gallium ion source was accelerated at 30 kV with a beam current that typically ranged from 120 pA to 4 nA depending on the milling size and speed. So, the power was 3.6 × 10^–6^ to 1.2 × 10^–4^ watt. Under these conditions, no ion beam damage was evident; any damage became noticeable only after imaging with the electron beam. The XPS spectra were fitted with Shirley-type background and Gaussian–Lorentzian convolution. The ratios of the Lorentzian and Gaussian components were not fixed during the peak fitting procedure. The elemental ratio of the surface was obtained by comparing the areas of the fitted curves divided by their atomic sensitivity factors of the system and normalized Pb intensity. The overall Pb signal was set to 1 as the reference because it was not expected to form a volatile compound that could readily leave the surface.

## Results and Discussion

The pristine MAPbBr_3_ single crystals were measured with XPS before CuPc passivation to check their initial surface composition. Their crystal quality has been confirmed with previous X-ray diffraction.^5,18,20^ The elemental ratios of C/N/Pb/Br are 1.53/1.08/1/2.82 and 1.31/1.29/1/2.50 for water and light exposures, respectively, where Pb’s ratio was set as 1 to be compared with. This ratio is close to the ideal stoichiometric value. The residual reactants used in the sample growth process could be responsible for the minor excess of carbon and nitrogen present, and the insufficient Br was probably due to Br vacancies on the surface. For each exposure, we did it with pristine and CuPc-passivated samples to study the effects of the CuPc passivation layer. It is worth noting that there is a variation in the initial peak positions of the two samples that shows the variations from cleave to cleave. However, these variations should not affect the observations on the dramatic effects of the CuPc layer during water and light exposures.

**Figure 1:**
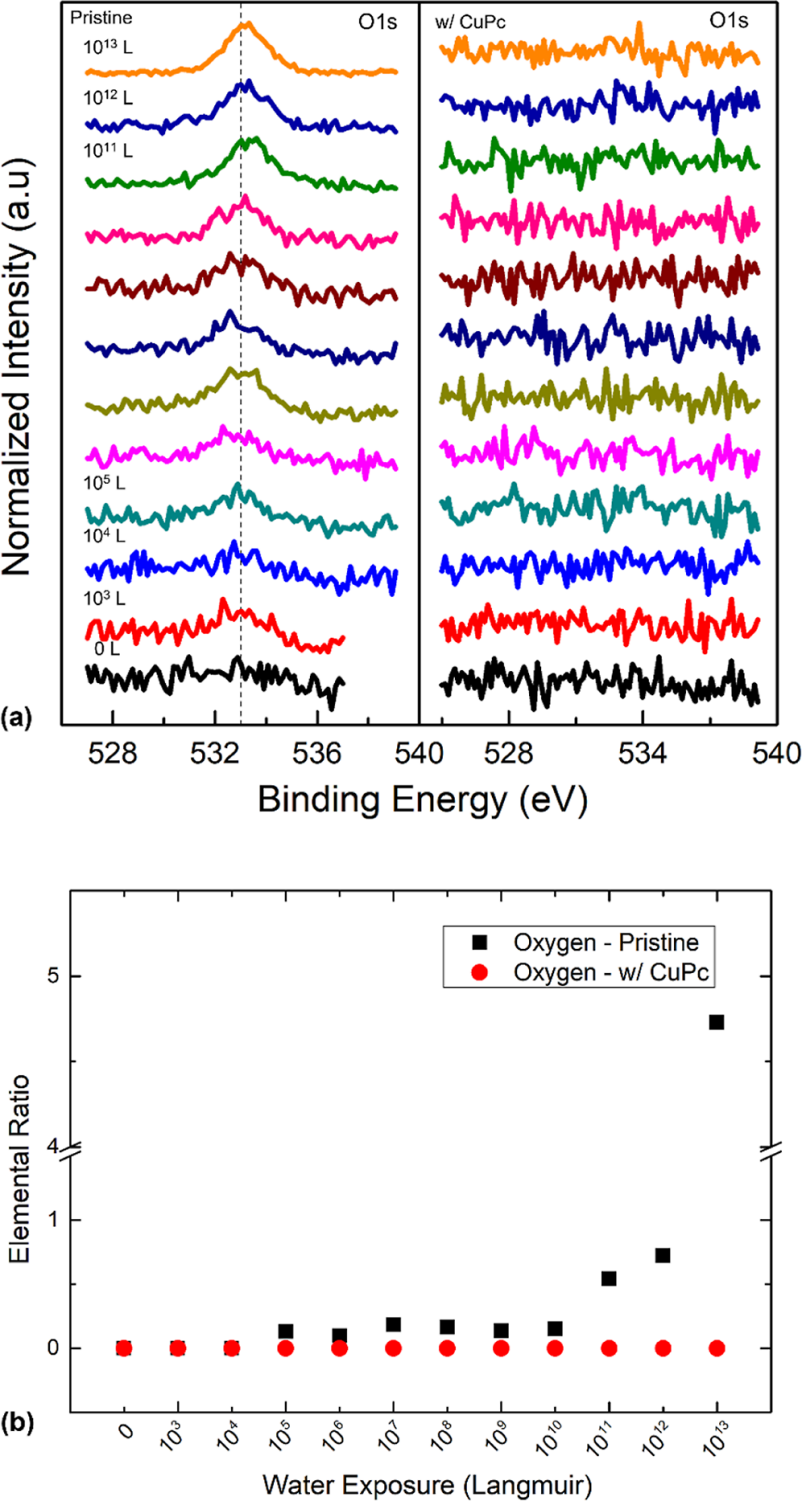
(a) Evolutions of the O 1s XPS spectra comparison of pristine and CuPc-passivated perovskites with increasing water exposure. (b) Oxygen ratio trend comparison for the pristine and the CuPc-passivated perovskites.

After the exposure to water, the most noticeable variation was observed in the oxygen spectra (Figure 1). Both samples had no oxygen prior to the water exposure, showing that the samples had good quality without contamination. On the pristine sample, detectable oxygen signals started appearing at 532.97 eV after 10^5^ L, and their intensity continued to increase with prolonged exposure. The peak had a BE shift toward the higher BE region and reached 533.22 eV after 10^13^ L. This 0.25 eV shift reflects an n-doping of the surface as the Fermi level moved closer to the conduction band minimum within the bandgap, showing that water acted as an n-dopant during the exposure. This observation is also consistent with our previous report.^20^ It eventually became the dominant signal on the surface with a ratio of 4.73, suggesting that the pristine surface had absorbed a considerable amount of water vapor. The water vapor was first physiosorbed on the pristine surface and then reacted with the perovskite, forming hydroxyl groups and hydroxide ions through water dissociation.^35^ Additionally, we learned that after 10^8^ L, water can react with perovskite, resulting in the loss of concentration of C, N, and Br.^20^ These elements become volatile species and escape from the surface. Also, water exposure caused significant roughening of the surface.^20^ In contrast, the CuPc-passivated sample did not show any trace of oxygen throughout the exposure. In our precisely controlled environment, the detected oxygen originated from water vapor, which means that water vapor could stick onto the surface of the pristine sample and keep accumulating, while the hydrophobic CuPc top layer could effectively repel the water from the surface and block the reactions between water and perovskite.

**Figure 2:**
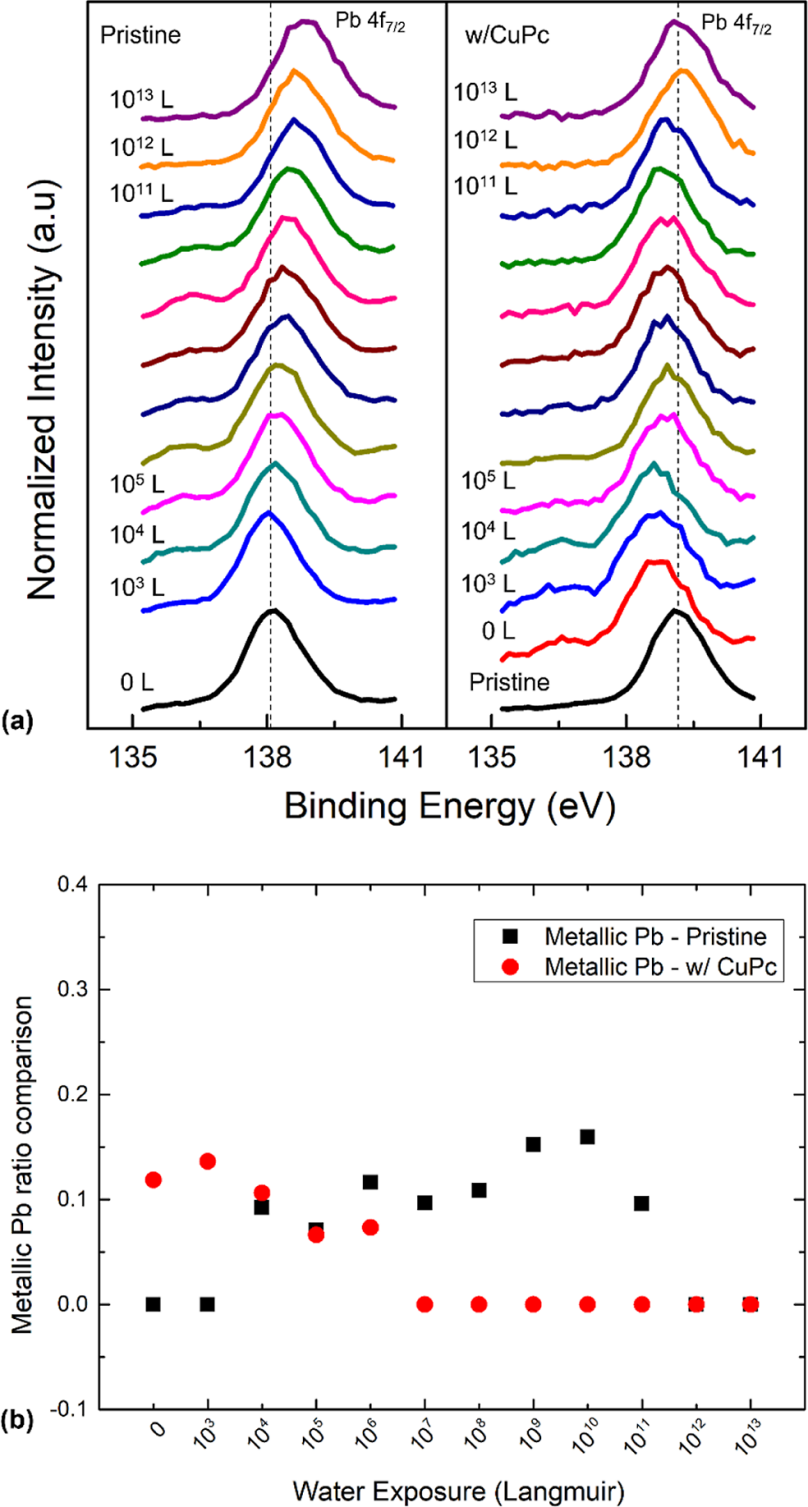
(a) Evolutions of Pb 4f_7/2_ XPS spectra comparison of pristine and CuPc-passivated perovskites with increasing water exposure. (b) Metallic Pb ratio trend comparison for pristine and CuPc-passivated perovskites with light exposure.

The XPS core level spectra and ratio comparison of Pb 4f are shown in Figure 2. Both samples saw a small secondary Pb peak formation during the exposure, which is attributed to the formation of metallic Pb.^18^ However, the formations emerged at different stages of the exposure on two samples. The pristine sample did not have the secondary peak when the exposure was initiated, but it showed up at 10^4^ L and continued to grow until 10^10^ L with a maximum ratio of 0.16, indicating the metallic Pb component made up 16% of the total Pb signal. The Pb 4f_7/2_ peak shifted from 138.13 to 138.81 eV after 10^13^ L of water exposure on a pristine sample, which suggests n-doping of the surface. X-rays could decompose up to 10% of the perovskite after 10 h of exposure.^20^ However, in this work, the X-ray-induced damage is expected to be insignificant as the XPS measurement time is about 20 min at each water exposure step. Then it quickly dropped and eventually vanished at 10^12^ L due to the reoxidation caused by the increasing water pressure. The transition of the Pb component and the decline of C, N, and Br concentrations confirm that the pristine sample was decomposed by the water exposure. On the CuPc-passivated sample, metallic Pb emerged before the exposure started. This can be explained by the deposition of the CuPc layer as thermal-evaporated CuPc thin films are reported to have the evaporation temperature above 300 °C.^36^ Therefore, the heated CuPc molecules could cause thermally induced degradation when they landed on the perovskite surface, resulting in the formation of metallic Pb. Different from the pristine sample, the metallic Pb component peaked at 10^3^ L and vanished at 10^7^ L on the CuPc-passivated sample. It shows that the thermal-induced degradation caused by hot CuPc molecules is limited and does little damage to the sample. Additionally, after the initial thermal degradation, water exposure did not further decompose the surface, which was attributed to the protection from the CuPc layer. Pd 4f XPS spectra also showed that the Pb peak shifted ∼0.45 eV toward the lower BE region after the CuPc passivation and then slowly moved back to the higher BE region with the water exposure. The BE movement suggests that the pristine surface was p-doped with CuPc and then gradually n-doped by water. This p-doping effect was also reported in other literature studies.^37–39^ The discussion above revealed that the CuPc top layer can protect the perovskite surface from water-induced degradation by effectively repelling the water molecules from the surface.

**Figure 3:**
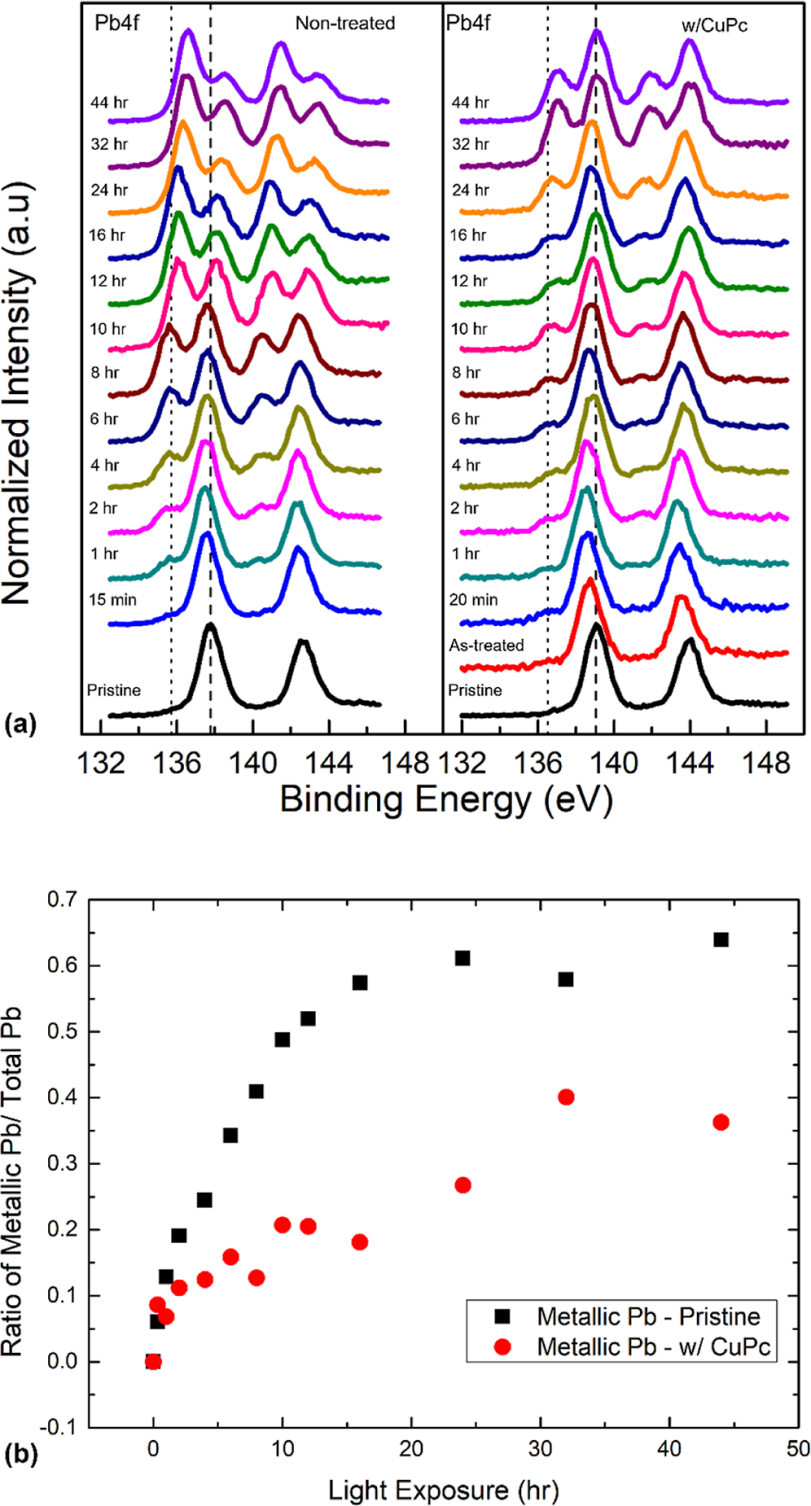
(a) Evolutions of Pb 4f XPS spectra comparison of pristine and CuPc-passivated perovskites with increasing light exposure. (b) Metallic Pb ratio trend comparison for pristine and CuPc-passivated perovskites with light exposure.

Light irradiation is another major challenge for perovskite stability, and we performed precisely controlled light exposure on CuPc-passivated perovskite to investigate its effect on the stability of the sample. Figure 3 shows a significant change, where the metallic Pb component is much larger than that observed during the previous water exposure. Both samples started to have metallic Pb peaks after the light exposure was initiated, indicating that the perovskite Pb decomposed into metallic Pb as a result of light-induced degradation. The metallic Pb quickly grew and became the dominant component after 10 h light exposure on the pristine surface and eventually made up 65% of the total Pb signal. In contrast, metallic Pb showed a much slower and milder growth on the CuPc-passivated surface with a final composition of 36% of the total Pb concentration. This value is equivalent to that of only 8 h of light exposure on the pristine surface. The perovskite Pb peak was located at 138.25 eV and began to shift toward the lower BE region when exposure started, indicating a p-doping of the pristine surface. This can be explained by either a decrease in the number of n-type traps on the surface due to photogenerated charge carriers or the reversible creation of p-type traps caused by light exposure.^40^ After 2 h of exposure, the peaks gradually shifted back to higher BEs and settled at 138.47 eV. The overall BE movement suggests n-doping of the pristine surface due to halide reduction and metallic Pb works as an n-dopant, which agrees well with the existing literature.^41,42^ The CuPc-passivated surface also showed a similar pattern; the difference is that the initial p-doping was mostly caused by CuPc. Pb and other core levels, such as Br and Ag (from the residual Ag paste for in situ cleavage), and VBM showed a ∼0.42 eV shift to the lower BE region, which is comparable to that in water exposure. Again, the consistent result confirmed that CuPc worked as a p-dopant.

**Figure 4:**
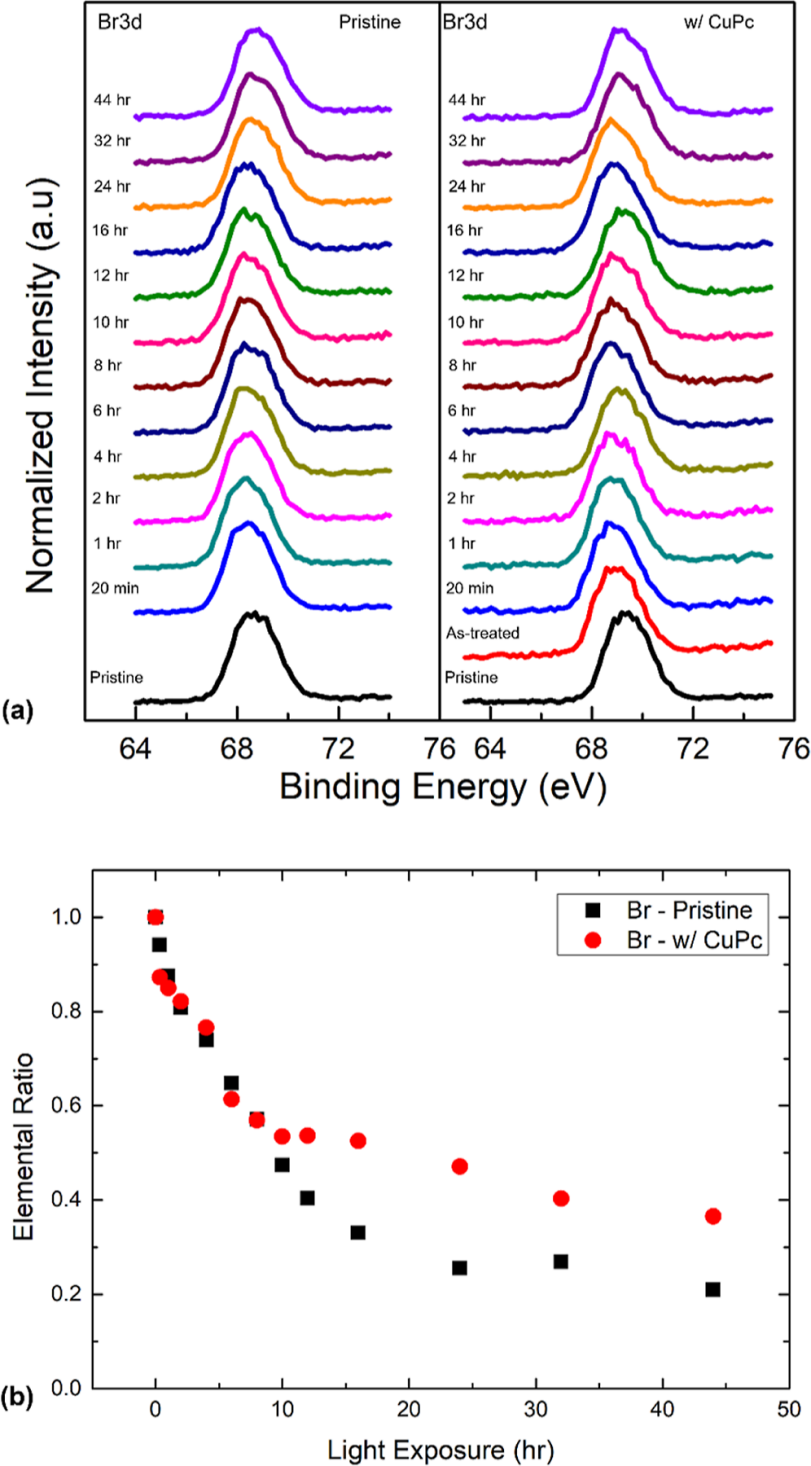
(a) Evolutions of Br 3d XPS spectra comparison of pristine and CuPc-passivated perovskites with increasing light exposure. (b) Br ratio trend comparison for pristine and CuPc-passivated perovskites with light exposure.

The decreasing trend in Br ratio, as shown in Figure 4b, indicates that the sample decomposed into a Br-containing volatile substance and left the surface.^20,43^ The final Br concentrations were 21 and 37% of their initial values for pristine and CuPc-passivated surfaces, respectively. The milder loss of Br on the CuPc-passivated surface suggests less severe degradation induced by light exposure. The concentration loss of perovskite C, N, and Br caused a metallic Pb rich surface which n-doped the surface. The presence of the CuPc passivation layer could inhibit the escape of decomposed volatile species and reduce the amount of light reaching the underlying perovskite surface by absorbing some of it. This effect impedes the degradation of perovskite Pb to metallic Pb and protects the surface from further damage. Furthermore, we believe that the degradation observed under CW laser illumination was primarily due to the light irradiation rather than the heating effect. This is supported by our previous demonstration that the MAPbBr_3_ single crystal could only be heated by 1–15 K with a CW laser, as calculated using Abbott’s heating model.^18^

**Figure 5:**
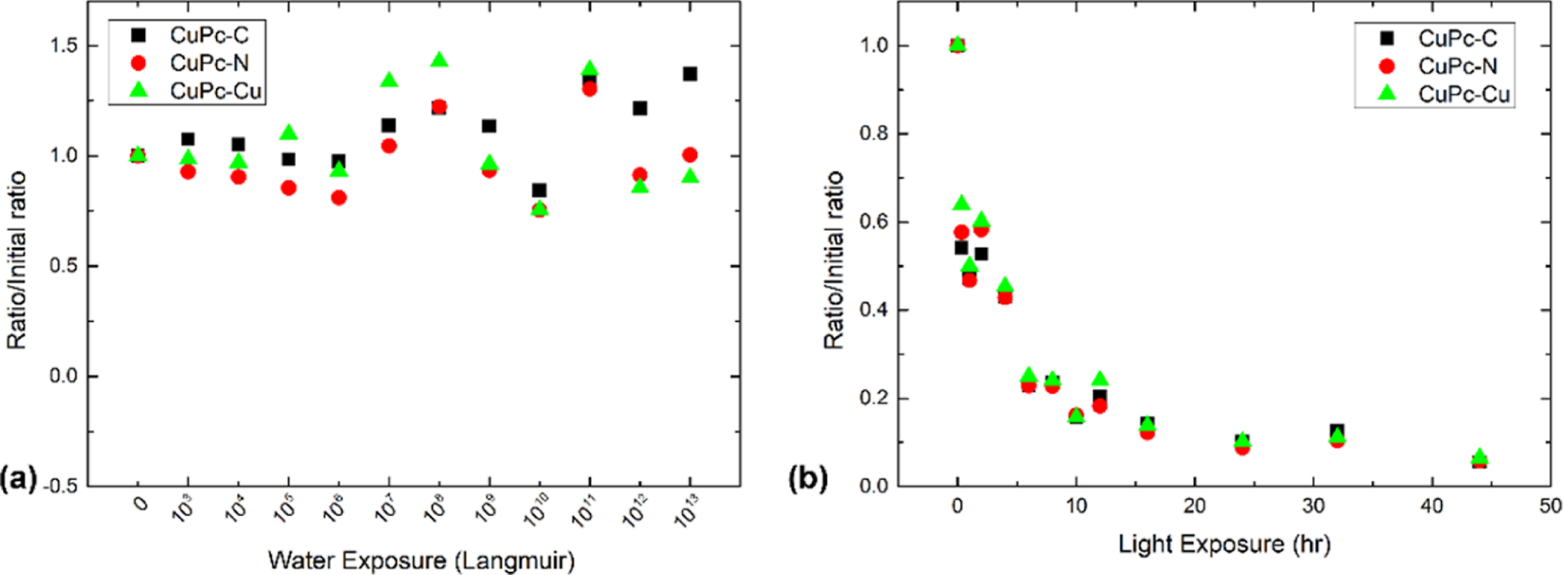
Carbon, nitrogen, and bromide ratio trend of the CuPc passivation layer for (a) water exposure and (b) light exposure.

By comparing the effects of water and light exposure, we observed that the CuPc passivation layer provided differing degrees of protection. While CuPc demonstrated its ability to shield the MAPbBr_3_ single crystal from water- and light-induced degradation, the elemental ratios of C, N, and Cu in CuPc exhibited distinctive behaviors. As shown in Figure 5, in the water-exposed sample, the ratios fluctuated as the level of water exposure increased. The N and Cu ratios remained similar to their initial values, while C gained approximately 37%, which is comparable to that on the pristine surface. This can be attributed to the formation of a hydrocarbon complex.^20^ This suggests that the CuPc top layer remained mostly intact, even after 12 days of water exposure in the UHV chamber. Surprisingly, in the light-exposed sample, all ratios quickly dropped as soon as the exposure started and eventually settled at approximately 5% of their initial value. This indicates that the light exposure caused laser desorption of the CuPc passivation layer, eventually exposing the underlying perovskite surface. The XPS core level evolution of C 1s, N 1s, and Cu 2p during water and light exposure can be found in Figures S1 and S2, respectively. There was no change in the spectral shape of the CuPc-related peaks, except for a decrease in peak intensity observed in the light-exposed sample due to the desorption of the CuPc layer by light. The peak position movements follow a pattern similar to that presented in the main text. The p-doping of the perovskite by CuPc is therefore mild enough and does not cause a chemical reaction. The comparison of XPS survey scans (Figure S3) confirms once again the different behaviors of the CuPc passivation layer after water and light exposures. In the water-exposed sample, the survey scans remained almost identical before and after the exposure as there were no new peaks or absence of peaks. In contrast, during light exposure, the survey scans for the as-treated and nonexposed regions were almost the same, while the light-exposed region exhibited the reappearance of Br and Pb signals from the underlying perovskite. This further confirms that laser irradiation caused the CuPc layer to be optically desorbed from the surface, which subsequently exposed the underlying perovskite. Figures 4b and 5b suggest that both perovskite degradation and the desorption of the CuPc layer occurred simultaneously after the light exposure started, which further indicates that the CuPc top layer could only slow down the light-induced degradation of MAPbBr_3_. The varying responses of the CuPc layer to different exposures result in differences in its protective performance on perovskites.

**Figure 6:**
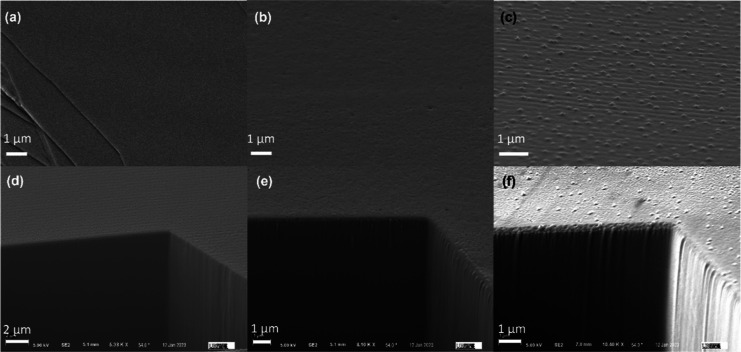
(a–c) SEM image of the surface of the freshly passivated, water-exposed, and light-exposed samples, respectively. (d–f) FIB-milled trenches for the freshly passivated, water-exposed, and light-exposed samples, respectively. The FIB-milled trench of the selected region revealed about 6–10 μm deep into the material.

We also conducted SEM and FIB measurements on our samples to investigate their morphological changes on both surfaces and bulk parts. As shown in Figure 6, the as-treated sample showed a clear and featureless surface, confirming that CuPc formed a smooth and uniform top layer via thermal evaporation. The FIB-milled trench showed that the bulk part of the sample was pristine to begin with. The morphological changes occurred after water and light exposure, but they were more moderate compared to these nonpassivated samples.^18^ The water-exposed sample demonstrated a mild-roughened surface, but the bulk part remained intact. The surface was still more uniform than that of the nonpassivated vacuum-exposed sample, which confirms that CuPc could protect the sample from water infiltration and the morphological change was merely due to degassing in the UHV chamber. For the light-exposed sample, small bumps formed across the surface. It could be attributed to metallic Pb aggregation. Even though its surface features were substantially smaller and more uniform than those of the nonpassivated sample.^18^ In contrast to the nonpassivated light-exposed sample, the CuPc-passivated sample did not exhibit the formation of cracks and voids in its bulk portion. This indicates that the top CuPc layer can prevent light penetration into the bulk and suppress volatile substances leaving the surface, thereby protecting the bulk part of the crystal. It is worth noting that not all hydrophobic organic materials can protect the underlying substrate. Rubrene, for example, is even worse at water protection as it cannot form a uniform layer (Figure S4). As a result, it repels water toward the exposed perovskite region, leading to an even higher water concentration on the perovskite surface, which ultimately results in more severe degradation.

## Conclusions

In this study, we examined the effects of the CuPc passivation layer on the MAPbBr_3_ single crystal under water and light exposures. Our findings indicate that a 40 Å thick CuPc top layer can effectively protect the perovskite against water exposure by repelling water molecules from the surface. However, it can only delay the light-induced degradation of perovskite by approximately 36 h. The differing protective abilities can be attributed to the distinct behaviors of the CuPc layer, which was found to be desorbed by light while remaining mostly intact after exposure to water. SEM and FIB results revealed that CuPc forms a uniform and featureless layer, resulting in significantly milder morphological changes on both the perovskite surfaces and bulk parts in comparison to those exposed to water and light without CuPc. Our investigation demonstrates that CuPc can not only improve the thermal stability of perovskites but also enhance their water and photostability, thereby expanding the methods for enhancing the stability of perovskites and contributing to the development of high-performance perovskite devices.

## Supporting Information Available

XPS evolution of C 1s, N 1s, and Cu 2p core levels of water exposure; XPS evolution of C 1s, N 1s, and Cu 2p core levels of light exposure; XPS survey scan comparison of as-passivated and end-stage surfaces for (a) water exposure and (b) light exposure; and SEM image of the MAPbBr_3_ surface with rubrene passivation after 10^9^ L water exposure (PDF)
