# Supplementary material for: CuPc Passivation of a MAPbBr3 Single Crystal Surface
Source: J Phys Chem C Nanomater Interfaces. 2023 Sep 26;127(39):19599–606. doi: 10.1021/acs.jpcc.3c04209 (PMC10561261; doi:10.1021/acs.jpcc.3c04209)
Supplement: Supplementary file 1 — jp3c04209_si_001.pdf [file jp3c04209_si_001.pdf]

CuPc Passivation of $MAPbBr_3$ Single Crystal Surface

Ke Wang[1], Benjamin Ecker[1], Mingze Li[2], Jinsong Huang[2], and Yongli Gao[1*]

[1]Department of Physics and Astronomy, University of Rochester, Rochester, NY 14627, United States

[2]Department of Applied Physical Sciences, University of North Carolina at Chapel Hill, Chapel Hill, NC, 27599, United States

* ygao@pas.rochester.edu

Supplementary Materials

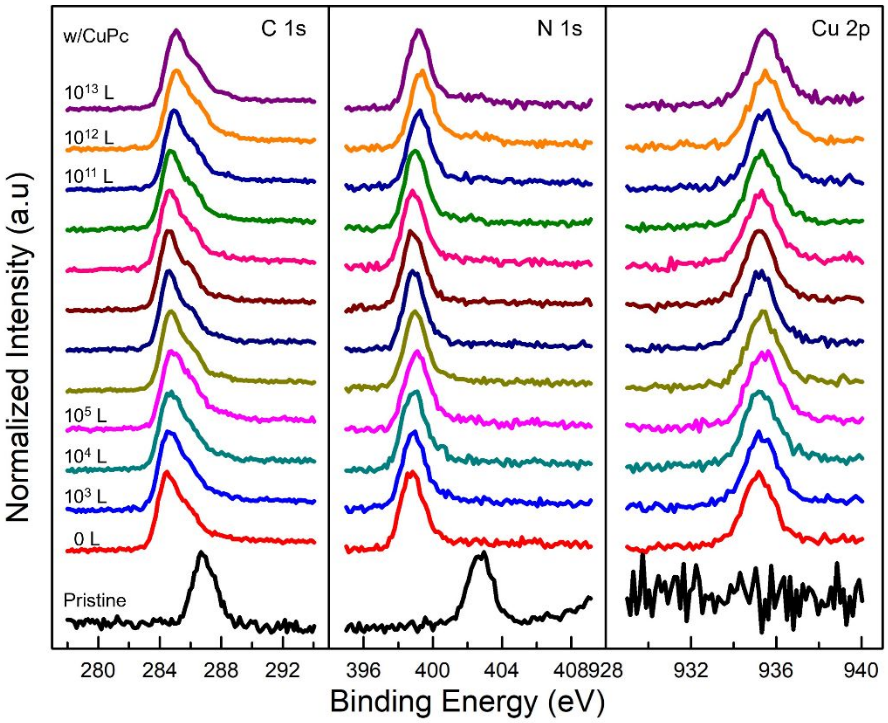

Figure S1. XPS evolution of C 1s, N 1s and Cu 2p core levels of water exposure.

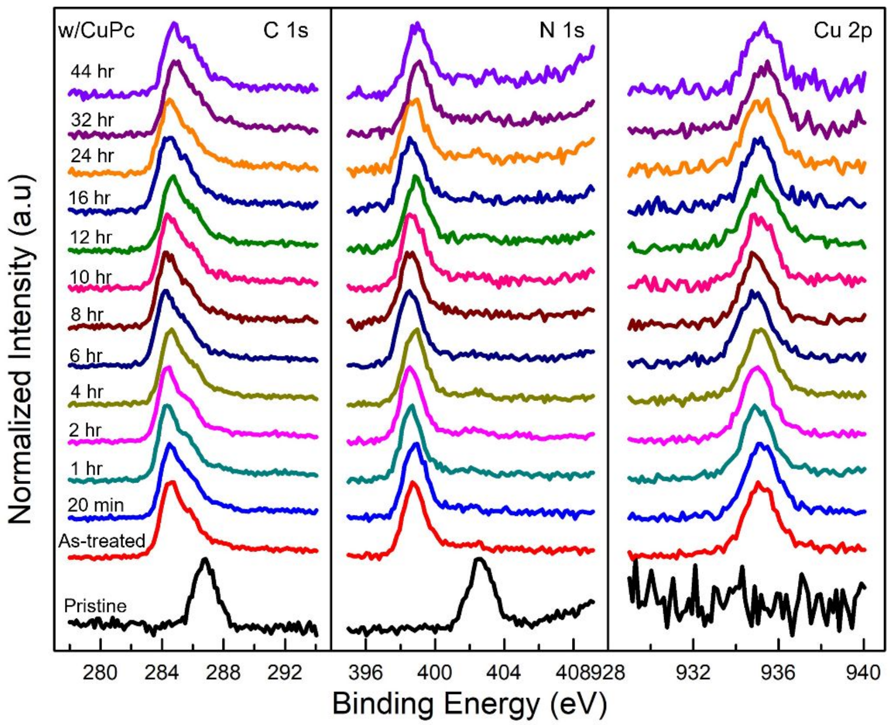

Figure S2. XPS evolution of C 1s, N 1s and Cu 2p core levels of light exposure.

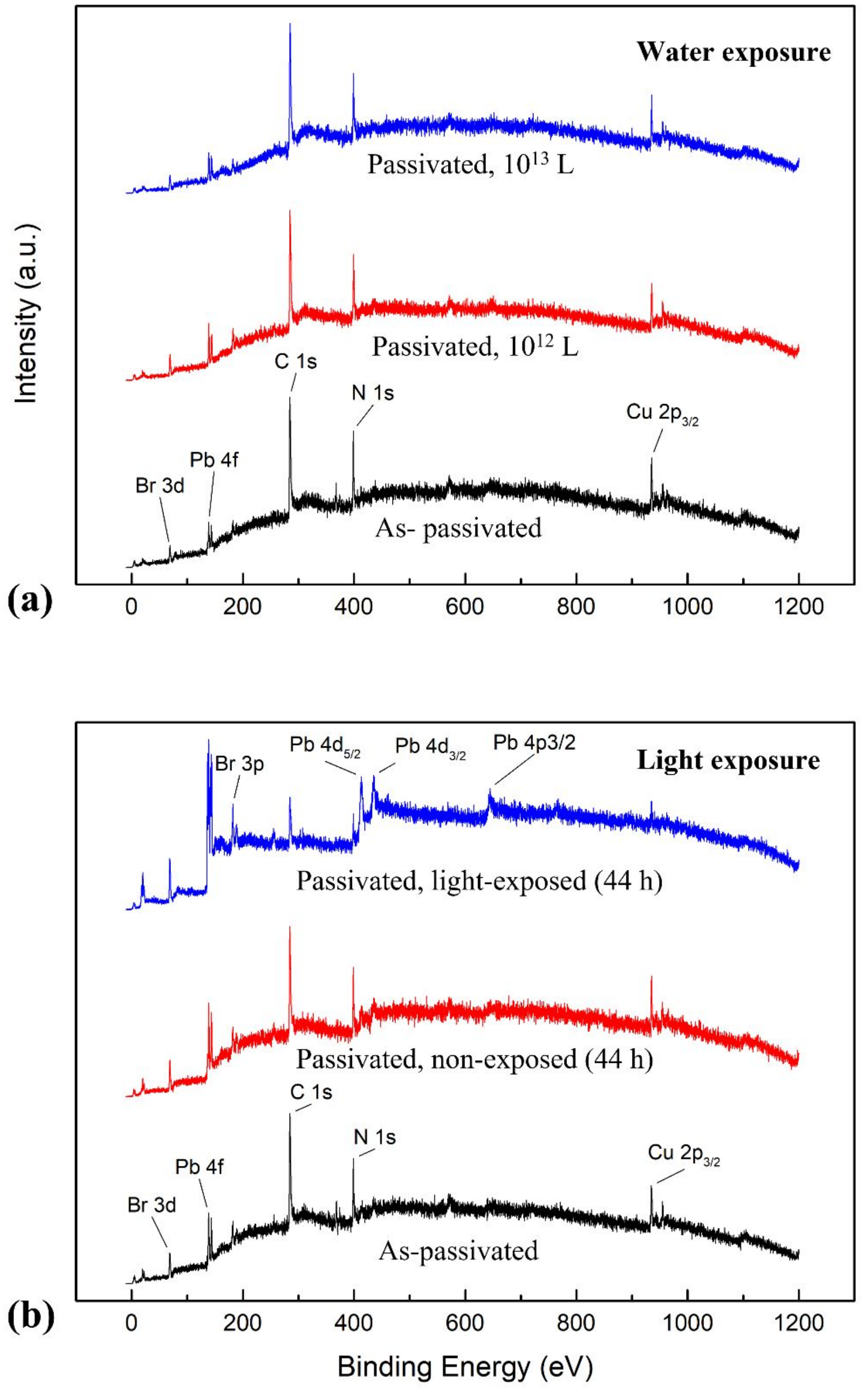

Figure S3. XPS survey scan comparison of as-passivated and end-stage surfaces for (a) water exposure and (b) light exposure.

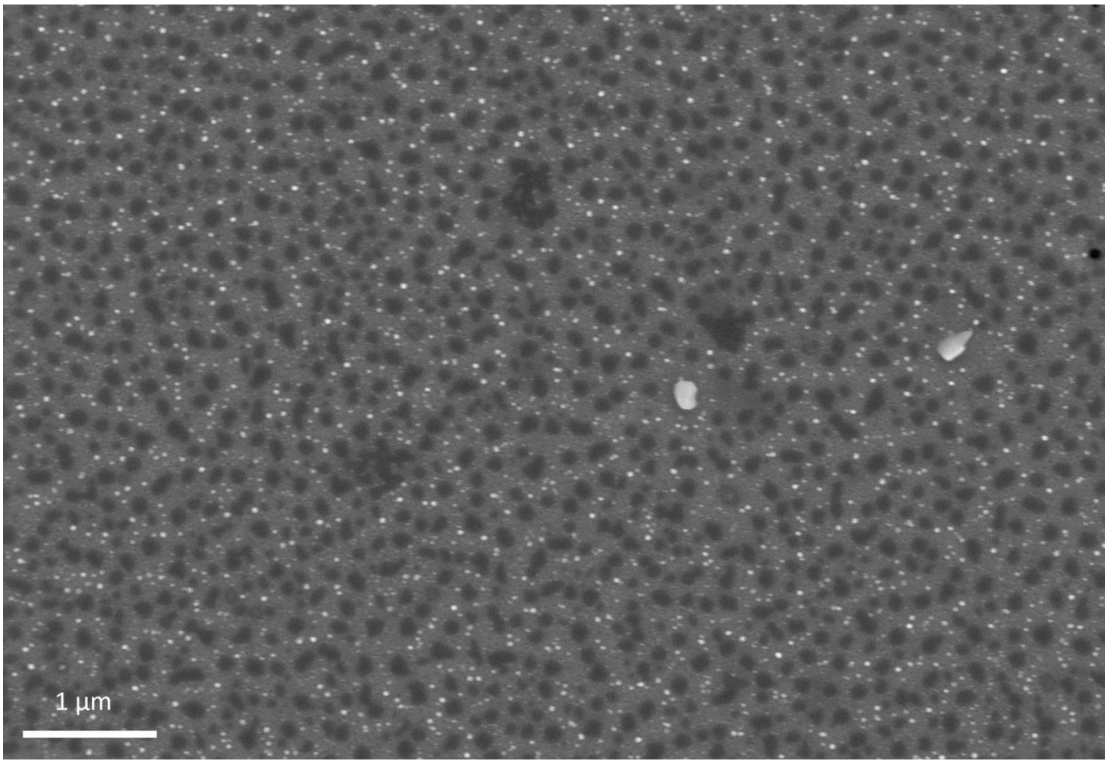

Figure S4. SEM image of $MAPbBr_3$ surface with rubrene passivation after 10^9 L water exposure.
